# Supplementary material for: Analysis of the Incidence of Type 2 Diabetes, Requirement of Insulin Treatment, and Diabetes-Related Complications among Patients with Cancer
Source: Cancers (Basel). 2023 Feb 8;15(4):1094. doi: 10.3390/cancers15041094 (PMC9953816; doi:10.3390/cancers15041094)
Supplement: Supplementary file 1 [file cancers-15-01094-s001.zip › cancers-2164156-supplementary.pdf]

Table S1. Description of time to event and censored data

	Number of diabetes event
**Event**	3294
Comparison	2319
Cancer	975
**Total censored (No event)**	21048
Comparison	13909
Cancer	7139
**Termination of study**	14735
Comparison	11172
Cancer	3563
**Loss to follow up / Drop-out**	6313
Comparison	2737
Cancer	3576

Table S2. Description of time to event and censored data according to the cancer type

	Number of diabetes event
**Event**	3294
Comparison	2319
GI tract	350
Liver	46
Gallbladder	21
Pancreas	16
Thyroid	68
Lung	53
Larynx	10
Lip, oral, pharynx	101
Breast	57
Kidney	22
Bladder	39
Prostate	50
Ovary	14
Uterus	41
Genital organ	4
Hematologic	21
Brain, CNS	7
Other and unspecified	55
**Total censored (No event)**	21048
Comparison	13909
GI tract	2188
Liver	669
Gallbladder	151
Pancreas	135
Thyroid	596

Lung	709
Larynx	83
Lip, oral, pharynx	482
Breast	538
Kidney	94
Bladder	156
Prostate	176
Ovary	134
Uterus	285
Genital organ	29
Hematologic	199
Brain, CNS	97
Other and unspecified	418
**Termination of study**	14735
Comparison	11172
GI tract	1063
Liver	139
Gallbladder	34
Pancreas	22
Thyroid	531
Lung	124
Larynx	41
Lip, oral, pharynx	305
Breast	418
Kidney	65
Bladder	83
Prostate	90
Ovary	98
Uterus	215

Genital organ	18
Hematologic	86
Brain, CNS	37
Other and unspecified	194
**Loss to follow up / Drop-out**	6313
Comparison	2737
GI tract	1125
Liver	530
Gallbladder	117
Pancreas	113
Thyroid	65
Lung	585
Larynx	42
Lip, oral, pharynx	177
Breast	120
Kidney	29
Bladder	73
Prostate	86
Ovary	36
Uterus	70
Genital organ	11
Hematologic	113
Brain, CNS	60
Other and unspecified	224

Table S3. Baseline characteristics of the study population according to the independent variables

Variables	Comparison (n = 16,2284)	Cancer (n = 8,114)	*P* value
**Sex**			1.000
Male	8496 (52.4%)	4248 (52.4%)	
Female	7732 (47.6%)	3866 (47.6%)	
**Ages (years)**			1.000
15-39	1784 (11.0%)	892 (11.0%)	
40-64	8444 (52.0%)	4222 (52.0%)	
>64	6000 (37.0%)	3000 (37.0%)	
**Residence**			1.000
Seoul	3390 (20.9%)	1695 (20.9%)	
Second area	3854 (23.7%)	1927 (23.7%)	
Third area	8984 (55.4%)	4492 (55.4%)	
**Household income**			1.000
Low (0–30%)	3580 (22.1%)	1790 (22.1%)	
Middle (30–70%)	5392 (33.2%)	2696 (33.2%)	
High (70–100%)	7256 (44.7%)	3628 (44.7%)	
**CCI**			1.000
0	9664 (59.6%)	4832 (59.6%)	
1	3408 (21.0%)	1704 (21.0%)	
≥2	3156 (19.4%)	1578 (19.4%)	

Comparison, subjects without Cancer; Seoul, the largest metropolitan area; second area, other metropolitan cities; third area, other areas; CCI, Charlson comorbidity index.

Table S4. Hazard ratios of incident diabetes by sex and age between comparison and cancer groups

**Sex**	**Male**		**Female**	
	Comparison	Cancer	Comparison	Diabetes
**Diabetes**				
Unadjusted HR (95% CI)	1.00 (ref)	1.31 (1.18-1.45)***	1.00 (ref)	1.15 (1.03-1.29)*
Adjusted HR (95% CI)	1.00 (ref)	1.35 (1.22-1.50)***	1.00 (ref)	1.22 (1.09-1.36)***

**Ages**	**15-39**		**40-64**		**>64**	
	Comparison	Cancer	Comparison	Cancer	Comparison	Cancer
**Diabetes**						
Unadjusted HR (95% CI)	1.00 (ref)	1.12 (0.68-1.83)	1.00 (ref)	1.38 (1.25-1.52)***	1.00 (ref)	1.15 (1.02-1.29)*
Adjusted HR (95% CI)	1.00 (ref)	1.16 (0.71-1.91)	1.00 (ref)	1.43 (1.29-1.58)***	1.00 (ref)	1.14 (1.01-1.28)*

HR, hazard ratio; CI, confidence interval; * $P<0.05$, ** $P<0.010$, and *** $P<0.001$.

Table S5. Subgroup analysis for incidence and risk of specific diabetes-associated complications in cancer patients

Variables	N	Case	Person year	Incidence rate	Unadjusted HR (95% CI)	Adjusted HR (95% CI)
**Diabetic nephropathy**						
Comparison	16228	138	146439.0	0.94	1.00 (ref)	1.00 (ref)
Cancer	8114	60	50144.3	1.20	1.29 (0.95-1.75)	1.37 (1.01-1.87)*
**Diabetic retinopathy**						
Comparison	16228	41	146863.0	0.28	1.00 (ref)	1.00 (ref)
Cancer	8114	8	50352.5	0.16	0.60 (0.28-1.30)	0.63 (0.29-1.34)
**Diabetic neuropathy**						
Comparison	16228	364	145464.8	2.50	1.00 (ref)	1.00 (ref)
Cancer	8114	129	49871.1	2.59	1.06 (0.86-1.29)	1.13 (0.93-1.39)
**Diabetic arthropathy**						
Comparison	16228	57	146734.6	0.39	1.00 (ref)	1.00 (ref)
Cancer	8114	14	50312.5	0.28	0.71 (0.40-1.27)	0.77 (0.43-1.38)

HR, hazard ratio; CI, confidence interval; * $P<0.05$, ** $P<0.010$, and *** $P<0.001$.
